# Magnesium-Assisted Cisplatin Inhibits Bladder Cancer Cell Survival by Modulating Wnt/β-Catenin Signaling Pathway

**DOI:** 10.3389/fphar.2021.804615

**Published:** 2022-01-27

**Authors:** Tianye Li, Zihan Tang, Chunting Li, Xiaoya Liu, Linglin Cheng, Zhijing Yang, Xiaojin Zhu, Weiwei Liu, Yongye Huang

**Affiliations:** ^1^ College of Life and Health Sciences, Northeastern University, Shenyang, China; ^2^ Department of Oral and Maxillofacia Surgery, Hospital of Stomatology, Jilin University, Changchun, China; ^3^ Jilin Provincial Key Laboratory of Tooth Development and Bone Remodeling, Changchun, China

**Keywords:** magnesium, cisplatin, β-catenin, cancer, autophagy

## Abstract

Magnesium, an essential mineral micronutrient, plays a role in the activation of various transporters and enzymes. The present study aimed to investigate the possibility of applying magnesium to enhance the efficacy of cisplatin which is still ranked as one of the major chemotherapeutic drugs for bladder cancer patients. Results showed that the survival rate and colony formation of bladder cancer cells were reduced by combinatorial treatment with cisplatin and magnesium chloride (MgCl_2_). The proportion of apoptotic cells was also increased in UC3 bladder cancer cells treated with a combination of cisplatin and MgCl_2_. Most importantly, a marked decrease in nuclear β-catenin was observed in cells that received cisplatin treatment. In addition, the nuclear β-catenin in cisplatin treated cells was further down-regulated by supplementing MgCl_2_. 6-bromoindirubin-3′-oxime (BIO), an inhibitor of glycogen synthase kinase-3 (GSK-3) that activates the Wnt/β-catenin signaling pathway by modulating β-catenin activity, was thus applied to further exploit the role of this signaling pathway in magnesium aided cancer treatment. The survival rate of bladder cancer cells was decreased by BIO treatment at concentrations of 1.0, 2.5 and 5.0 μM accompanied by increased β-catenin expression. However, the expression of β-catenin in MgCl_2_-treated cells was lower than in untreated cells under the same BIO concentration. The expression of cleaved caspase-3, cleaved caspase-9 and microtubule-associated protein 1 light chain 3- II (LC3-II) was highest in cells treated with MgCl_2_ and 5.0 μM BIO among the examined groups. Our findings reveal that magnesium could contribute to cisplatin-based chemotherapy by moderately regulating the Wnt/β-catenin signaling pathway.

## 1 Introduction

Bladder cancer is the most frequently diagnosed urinary system malignancy with an estimated 573,278 new cases and 212,536 deaths worldwide in 2020 (Sung et al., 2021). In recent years, a large body of research has focused investigating on the pathogenesis of bladder cancer, identifying novel molecular therapeutic targets, and evaluating therapeutic. Although great advances have been made in the management of bladder cancer, there are still undefined molecular mechanisms that influence the therapeutic outcomes of this disease.

Over the past few decades, cisplatin has been one of the most widely used first-line chemotherapeutic drugs for the treatment of various solid tumors, including lung, ovarian and bladder cancer (Köberle and Schoch, 2021; Tchounwou et al., 2021). Despite positive clinical outcomes, substantial side effects or drug resistance development have been reported in many studies of cisplatin monotherapy. Alternatively, platinum-based combination chemotherapy is considered the preferred initial therapy for bladder cancer (Kwon and Seo, 2021). The action of magnesium as an essential mineral micronutrient may lead to it playing a unique role in both cancer development and therapy. In our previous work, we found that treatment with moderate amounts of magnesium could enhance the inhibitory effect of valproic acid on cell proliferation in bladder cancer (Li et al., 2020). Therefore, the present study aimed to investigate the possibility of applying magnesium treatment to promote the anti-tumor effects of cisplatin.

The Wnt/β-catenin signaling pathway is a canonical pathway of signal transduction in physiological and pathological processes. This signaling pathway is intimately associated with various biological processes, including embryonic development, proliferation, apoptosis, and cell cycle distribution (Zhang and Wang, 2020; Sidrat et al., 2021). The aberrant regulation of the Wnt/β-catenin cascade leads to the development and progression of cancer. Wnt signaling is controlled by various secreted Wnt glycoproteins that function via autocrine and paracrine pathways in mammalian cells (Willert et al., 2003; Komiya and Habas, 2008). Currently, 19 Wnt genes belong to the coiled family of transmembrane receptors and act as the coreceptor for low-density-lipoprotein-related protein5/6 (LRP5/6) (Lee et al., 2012; Lorzadeh et al., 2021). The central molecule in this pathway is the cytoplasmic protein β-catenin that controls the switch of the Wnt signaling pathway by precisely regulating its level in the cytosol (Anastas and Moon, 2013). Under circumstances where the Wnt signaling is lacking, cytosolic β-catenin would be actively phosphorylated and degraded *via* a destruction complex composed of glycogen synthase kinase 3α/β (GSK 3α/β), adenomatous polyposis coli (APC), casein kinase 1α/δ (CK1α/δ), axin, and protein phosphatase 2A(PP2A). In contrast, the stabilization and accumulation of β-catenin would give rise to its transportation in to the nucleus, promoting the expression of target genes (Nusse and Clevers, 2017). Aberrations in Wnt/β-catenin signaling are shown to be closely associated with bladder carcinogenesis (Chehrazi-Raffle et al., 2021). Possibly, the correction and/or regulation of Wnt/β-catenin components would be helpful to the bladder cancer therapy. Modulating the activation of the Wnt/β-catenin axis has been suggested to promote sensitivity to cisplatin in several types of cancers (Jiang et al., 2019; Wang et al., 2020). Furthermore, Wnt/β-catenin signaling is shown to be involved in magnesium treatment in many cell models (Montes de Oca et al., 2014; Pan et al., 2017). Thus, regulating Wnt/β-catenin signaling could contribute to cisplatin-based cancer therapy in the context of adjusting magnesium abundance.

## 2 Materials and Methods

### 2.1 Drugs, Chemicals and Antibodies

Cisplatin (cis-diamminedichloroplatinum II) with purity >98% was purchased from Sigma-Aldrich, and a stock solution of cisplatin at a concentration of 2.5 mg/ml (8.33 mM) was prepared in water and stored at −20°C. Magnesium chloride (MgCl_2_) and magnesium sulfate (MgSO_4_) were purchased from Sigma-Aldrich, and the stock solutions (0.70 mol/L magnesium, 1,000×) was prepared in Dulbecco’s modified Eagle’s medium (DMEM) and stored at −20°C. The treatment concentration and duration were according to our previous studies (Li et al., 2020). 6-bromoindirubin-3′-oxine (BIO) was purchased from Selleck. The BIO powder was dissolved in DMSO to produce a 10 mM stock solution and stored at −20°C. All the stock solutions were diluted in culture medium immediately prior to experimentation. Unless otherwise specified, all other chemicals were purchased from Sigma-Aldrich.

The primary antibodies to voltage-dependent anion channel (VDAC1), Bip, vimentin, p-p38, p38, β-catenin, Wnt family member 5A (Wnt5A), caspase-3, Lamin A/C, β-actin and glyceraldehyde-3-phosphate dehydrogenase (GAPDH) were provided by Cell Signaling Technology. The antibodies to cyclin dependent kinase 1 (CDK1), microtubule associated protein 1 light chain 3 (LC3), DNA damage inducible transcript 3 (CHOP or DDIT3) and zona occludens 1 (ZO-1) were provided by Proteintech. The antibodies to p-c-Myc, c-Myc, Bcl-2, Bak, cyclin B1, p21, mixed lineage kinase domain-like (MLKL) and caspase-9 were provided by Bimake. The antibodies to Bid was provided by Signalway Antibody, and the antibody to Bax was provided by Thermo Scientific.

### 2.2 Cell Culture

Human bladder cancer cell lines (UM-UC3, UM-UC5, and HEK 293) were cultured in DMEM containing 10% fetal bovine serum (FBS), 1% nonessential amino acid, 1% glutamine, and 1% penicillin-streptomycin (100 U/ml penicillin and 100 μg/ml streptomycin) and maintained in a 37°C incubator with 5% CO_2_ atmosphere.

For the treatment of bladder cancer cells, moderate amounts of cells were seeded into dishes or plates. After overnight adherent, control group was replaced with fresh complete culture medium, and MgCl_2_-, cisplatin-, MgCl_2_ combined with cisplatin treated groups were replaced with fresh complete culture medium contain 42 mM MgCl_2_, 8.33 μM cisplatin and 42 mM MgCl_2_ combined with 8.33 μM cisplatin for another 24 h, respectively. Unless otherwise specified, all other treatments were as described above.

### 2.3 Cell Viability Assay

UC3 and UC5 cells were seeded into 96-well plates at a density of 1 × 10^4^ cells/well, treated with 200 μl of different concentration of culture medium containing MgCl_2_ (42 mM) and/or cisplatin (1.67, 3.33, 8.33, or 16.66 μM) and cultured at 37°C in a 5% CO_2_ atmosphere. After different time duration (24, 48 or 72 h), 10 μl Cell Counting Kit-8 (CCK-8) solution mixed with 100 μl culture medium was added to each well. The cells were then incubated for 1.5 h at 37°C. Absorbance (OD) was measured using a microplate reader at 450 nm wavelength. Data are represented as the mean of five replicates.

### 2.4 Propidium Iodide Staining

Morphological evaluation of cells treated with MgCl_2_, cisplatin, or BIO alone and in combination was done *via* PI staining. In brief, cells were seeded into a 24-well plate at a density of 5 × 10^4^ cells/well and treated with MgCl_2_ (42 mM) and/or cisplatin (8.33 μM) and/or BIO (0.5, 2.5, or 5 μM) for 24 h. Then, the cells were washed with PBS and treated with 200 µl PI (1 μg/ml) for 5 min at room temperature in the dark. Finally, the cells were assessed and photographed under a fluorescence microscope (Leica, Shanghai, China). The cell death rate (%) was calculated as the proportion of PI positive cells.

### 2.5 Clone Formation Assay

For each well, 1.5 ml culture medium with 0.6% agar was used as the bottom gel and coated into 6-well plates. After solidification, 1 × 10^3^ treated cells were suspended in 1 ml medium with 0.35% agar and poured onto the bottom gel. A volume of 150 μl culture medium was added into these plates every 2 days to prevent the cells from drying. Each group had three replicates. After 20 days of incubation, the cells were washed with PBS, fixed with 4% paraformaldehyde for 30 min, and stained with 0.1% crystal violet solution for 15 min. The stained colonies were counted and photographed for further analysis.

### 2.6 Cell Apoptosis Analysis by Flow Cytometry

Cell apoptosis was assessed by flow cytometry using an annexin V-FITC/PI (Fluorescein Isothiocyanate, FITC) apoptosis detection kit. After being incubated with MgCl_2_ and/or cisplatin for 24 h, cells were digested with 0.25% trypsin and collected by centrifugation. The cells were then washed twice with cold PBS and resuspended with 100 μl of 1× binding buffer containing 5 μl of annexin V-FITC/PI, mixing gently. After treatment for 15 min in the dark at room temperature, 400 μl of 1× binding buffer was added to the labeled cells and the cells were immediately analyzed using a flow cytometer.

### 2.7 Cell Cycle Analysis by Flow Cytometry

Cell cycle analysis was performed as previous described (Huang et al., 2021a). Cells were treated with MgCl_2_ (42 mM) and/or cisplatin (1.67, 3.33 or 8.33 μM) for 24 h, collected using 0.1% trypsin in 2.5 mmol/L EDTA, and fixed with 70% ethanol at −20°C overnight. Then, cells were stained with PI/RNase Staining Buffer Solution and analyzed using a flow cytometer.

### 2.8 Real-Time Quantitative Polymerase Chain Reaction

qPCR was used to analyze the mRNA levels in the bladder cancer cells. Total RNA was isolated using the TRIzol reagent (Tiangen Biotech) according to the manufacturer’s instructions. The All-in-One cDNA Synthesis SuperMix kit (Bimake, Houston, TX, United States) was used to reverse transcribed 2 μg total RNA into complementary DNA (cDNA) according to the manufacturer’s instructions. qPCR was performed with 2× SYBR Green qPCR Master Mix (Bimake, Houston, TX, United States) following the manufacturer’s protocol on a CFX96 real-time PCR detection system. GAPDH was used as a reference gene, and primer sequences were listed in Supplementary Table S1. Based on the analysis of the amplification and melting curves, the relative expression of the target genes was calculated using the 2^−ΔΔCt^ method.

### 2.9 Western Blot Analysis

Following treatment with MgCl_2_ and/or cisplatin for 24 h, cells were washed twice with PBS and the total protein was harvest using a lysis buffer with phenylmethanesulfonyl fluoride (PMSF). After centrifugation, the protein contents were quantitated using the bicinchoninic acid (BCA) protein assay kit (Beyotime, Shanghai, China). Equal amounts (20 μg) of cell extracts were separated by 12% sodium dodecyl sulfate-polyacrylamide gel electrophoresis (SDS-PAGE), and transferred to a polyvinylidene fluoride (PVDF) membrane, blocked with a 5% non-fat milk solution for 1 h at room temperature, and incubated with primary antibodies overnight at 4°C. The membranes were then washed thrice with TBST and incubated with the appropriate horseradish peroxidase (HRP)-conjugated secondary antibody at room temperature for 1 h. After being washed thrice with TBST, the protein bands were visualized using an electrochemiluminescence (ECL) detection system. To ensure equal loading, blots were incubated with β‐actin or GAPDH antibodies as a loading control.

### 2.10 Migration Detection

Cell migration was measured by a 24-well trans-well chamber (8 µm pore size, Greiner Bio-one). After 24 h of MgCl_2_ and/or cisplatin treatment, 2 × 10^4^ cells suspended in 100 μl serum-free DMEM were seeded into the upper chamber of each well, and 600 μl medium containing 2.5% FBS was added to the lower chamber. After a 24 h incubation, the chambers were washed thrice with PBS to wash away the cells remaining in the upper membrane while cells that migrated were fixed in methanol for 30 min, stained with 0.1% crystal violet for 15 min at room temperature and viewed under a microscope at 200× magnification.

### 2.11 Separation of Nuclear and Cytoplasmic Proteins

For the detection of β-catenin distribution, cytoplasmic and nuclear proteins were isolated using the Nuclear and Cytoplasmic Protein Extraction Kit (Beyotime, Shanghai, China), according to the manufacturer’s instructions. Briefly, MgCl_2_ and/or cisplatin treated cells were washed twice with precooled PBS, then harvested with an appropriate amount of cytoplasmic protein extraction reagent including PMSF. The cell suspension was centrifuged at 12,000 ×g at 4°C for 5 min, and the supernatant was collected as the cytoplasmic protein fraction. The nuclear protein extraction reagent including PMSF was added into the pellet, and the solution was intensely vortex-mixed every 5 min for a total of 30 min. Following centrifugation at 12,000 ×g at 4°C for 10 min, the nuclear proteins in the supernatant were collected. The protein concentration was measured by a BCA protein assay kit (Beyotime, Shanghai, China). Western blotting analysis was used to detect β-catenin abundance in the cytoplasmic and nuclear fractions.

### 2.12 Immunofluorescence and Nuclear Staining

To determine β-catenin nuclear translocation, bladder cancer cells were seeded at 5 × 10^4^ cells per well into 24-well plates and treated with MgCl_2_ and/or cisplatin for 24 h. The cells were rinsed with PBS and fixed with 4% paraformaldehyde for 30 min, permeabilized with 0.2% Triton X-100 for 30 min, and blocked in 1% bovine serum albumin (BSA) in PBS for 30 min at room temperature. The fixed cells were then incubated with primary anti-β-catenin antibody at 4°C overnight. After that, cells were rinsed thrice with 0.2% Tween-20 in PBS and incubated with the appropriate fluorescence‐conjugated secondary antibody at 4°C overnight in the dark. The nuclei were stained with Hoechst33342 for 5 min. Finally, the cells were examined with a fluorescence microscope (Leica, Shanghai, China).

### 2.13 Statistical Analysis

Data were expressed as mean ± standard error of the mean (SEM). The statistical analyses were conducted using SPSS and GraphPad prism software. Multiple comparisons for differences were conducted by one-way ANOVA and the differences between the two groups were assessed using the unpaired Student’s t-test. A *p*-value < 0.05 was considered statistically significant. Each experiment was repeated three times.

## 3 Results

### 3.1 Magnesium Increases Cisplatin-Induced Inhibition of Human Bladder Cancer Cell Proliferation

**FIGURE 1:**
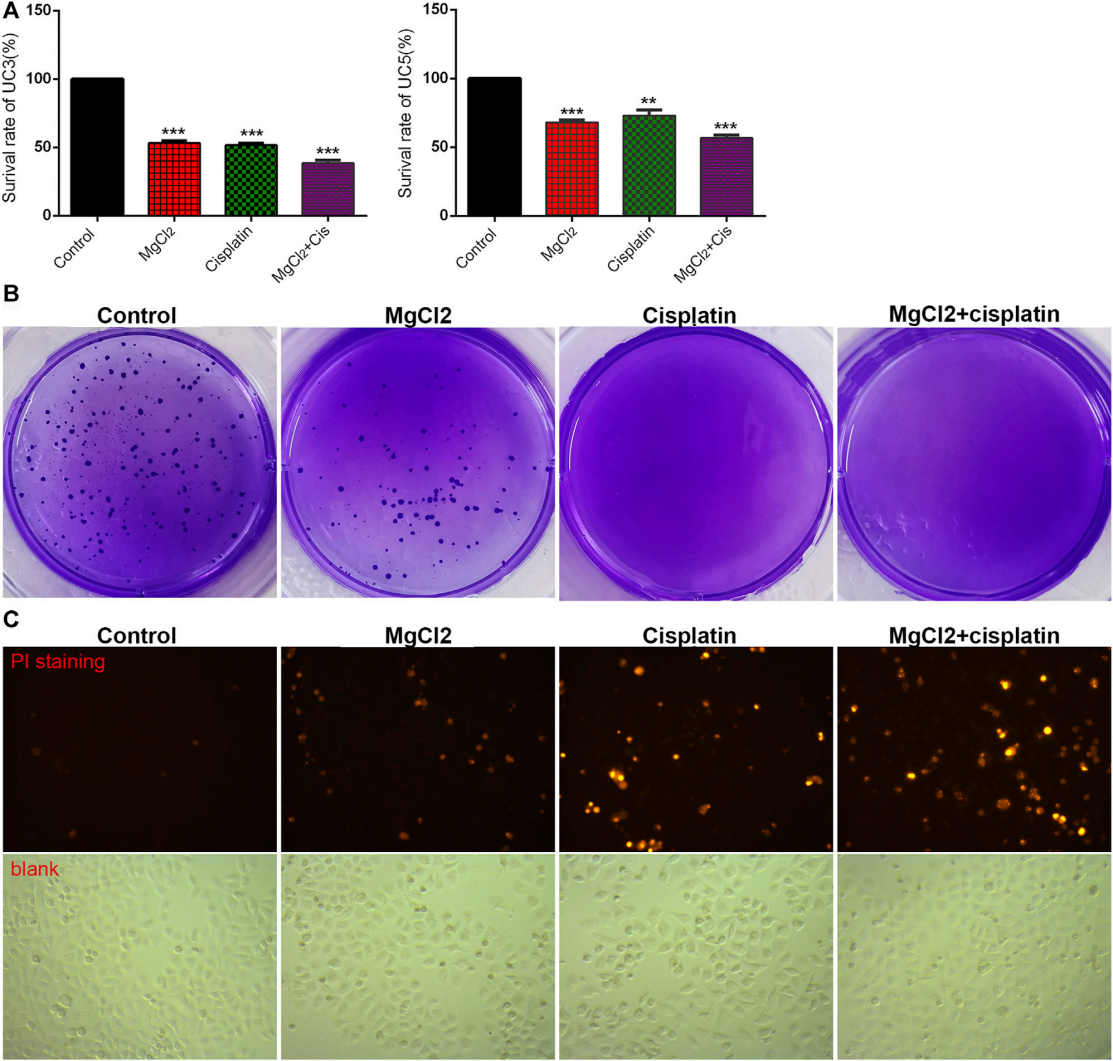
Combinatorial treatment with MgCl_2_ and cisplatin (Cis) suppresses the survival of bladder cancer cells. **(A)** The cell survival of MgCl_2_ and/or cisplatin treated UC3 and UC5 bladder cancer cells for 24 h was examined by the CCK-8 assay. **(B)** The colony formation of UC3 cells treated with MgCl_2_ and/or cisplatin. The results of quantification were presented in Supplementary Figure S1B. **(C)** PI staining of UC3 cells treated with MgCl_2_ and/or cisplatin for 24 h. The immunofluorescence intensity of PI staining was showed in Supplementary Figure S1C.

To determine the role of magnesium in cisplatin therapy for bladder cancer, cytotoxicity was first examined via the CCK-8 assay in UC3 and UC5 cells. Compared with the control, the survival rate in cisplatin- and MgCl_2_-treated UC3 cells was 51.6 ± 1.4% and 53.2 ± 1.5%, respectively (Figure 1A; Supplementary Figure S1A). In addition, the survival rate of UC3 cells was further decreased by combinatorial treatment with cisplatin and MgCl_2_ (38.5 ± 2.2%, *p* < 0.001). In UC5 cells, the survival rate in cells received cisplatin or MgCl_2_ treatment alone was 73.1 ± 4.0% and 68.0 ± 1.9%, respectively. Similar to UC3 cells, MgCl_2_ increased the inhibition of UC5 cell proliferation by cisplatin, with a survival rate of 56.7 ± 2.2% (*p* < 0.001). To confirm the role of magnesium in anti-tumor function, MgSO_4_ was also applied to treat UC3 cells. It is found that high concentration of MgSO_4_ alone, or in combination with cisplatin could significantly inhibit the survival of UC3 cells (Supplementary Figure S2). Furthermore, high concentration of both MgCl_2_ and MgSO_4_ could also prevent the survival of HEK 293 cells. Considering its inhibitory effect on cell proliferation, UC3 cells were mainly used to further uncover the role of magnesium in cancer combinatorial therapy in subsequent experiments. The results of the colony formation assay showed a lower colony number in the MgCl_2_-treated group than the control group, with nearly no colony formation in the cisplatin group and combinatorial treatment group (Figure 1B). In sum, both magnesium and cisplatin showed an inhibitory effect on cell proliferation.

**FIGURE 2:**
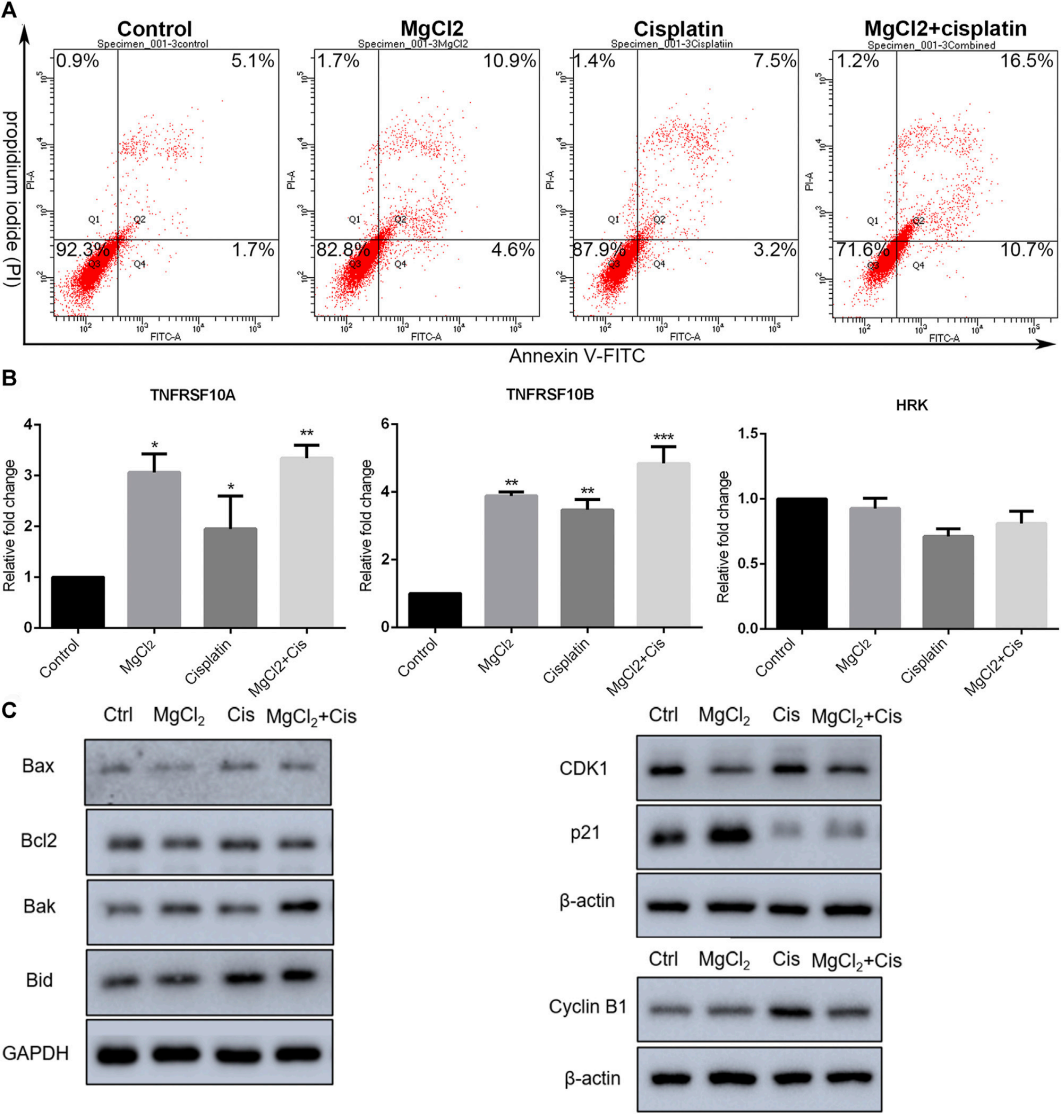
Combinatorial treatment with MgCl_2_ and cisplatin (Cis) induces apoptosis in UC3 bladder cancer cells. **(A)** Apoptosis in bladder cancer cells treatment with MgCl_2_ and/or cisplatin for 24 h was determined by annexin V-FITC/PI staining using flow cytometry (also seen Supplementary Figure S1D). **(B)** Expression of apoptosis-related genes as determined by qPCR (mean ± SEM of duplicate experiments). *p < 0.05 vs. control, **p < 0.01 versus control, and ***p < 0.001 vs. control. **(C)** Expression of genes associated with apoptosis and cell cycle distribution as measured by western blot analysis. Densitometry of western blot was showed in Supplementary Figure S5.

The inhibition of cell proliferation is always accomplished through the induction of cell death. As revealed by PI staining, there was nearly no cell death in the control group, and MgCl_2_ or cisplatin treatment alone induced low levels of cell death in both UC3 and UC5 bladder cancer cells (Figure 1C; Supplementary Figure S3). As expected, MgCl_2_ treatment exacerbated the cell death induced by cisplatin. The annexin V FITC/PI staining assay was performed and assessed by flow cytometry to further evaluate the occurrence of cell death. As shown in Figure 2A, the UC3 cells survival rate in the control group, MgCl_2_ group, and cisplatin group was 92.3, 82.8 and 87.9%, respectively. Cells that received both MgCl_2_ and cisplatin treatment showed the lowest survival rate (71.6%) among the examined groups (*p <* 0.001). The apoptosis rate in the control group, MgCl_2_ group, cisplatin group and combined group was 6.8, 15.5, 10.7 and 27.2%, and the necrosis rate in these four groups was 0.9, 1.7, 1.4 and 1.2%, respectively. Similarly, combinatorial treatment with MgCl_2_ and cisplatin enhanced the apoptosis induction of UC5 bladder cancer cells (Supplementary Figure S4A). The expression of apoptosis-related genes was also determined by qPCR and western blotting. The results of the qPCR analysis indicated that the expression of TNFRSF10A and TNFRSF10B was significantly up-regulated in cells subjected to combinatorial treatment with MgCl_2_ and cisplatin, but there was no significant change in HRK expression (Figure 2B). The ratio of Bax/Bcl2 in cells treated with the combination of MgCl_2_ and cisplatin was highest among the examined groups as revealed by the western blot analysis (Figure 2C; Supplementary Figure S4B). In addition, the expression of Bak and Bid was also shown to be increased by the combinatorial treatment, further confirming that magnesium could be conducive to enhancing the antitumor effects of cisplatin.

The literatures have also showed involvement of cell cycle arrest in the inhibition of proliferation (Sun et al., 2021). The expression of CDK1, cyclin B1, and p21 was examined by western blotting. As shown in Figure 2C; Supplementary Figure S4C, the expression of cyclin B1 was enhanced by combinatorial treatment with cisplatin and MgCl_2_ in comparison with the control. In contrast, the expression of p21 was down regulated in cells that received both cisplatin and MgCl_2_ treatment. Flow cytometry was conducted to determine the cell cycle distribution under combinatorial treatment. Results indicate that cells treatment with MgCl_2_ were caused G0/G1 arrest, while cisplatin caused inhibition of S-phase. Magnesium-enforced bladder cancer cell death induced by cisplatin is associated with the integration regulation of cell cycle distribution including G0/G1 arrest and inhibition of S-phase compared with control (Supplementary Figure S5A).

### 3.2 Magnesium in Combination with Cisplatin Disturbs Cellular Homeostasis

**FIGURE 3:**
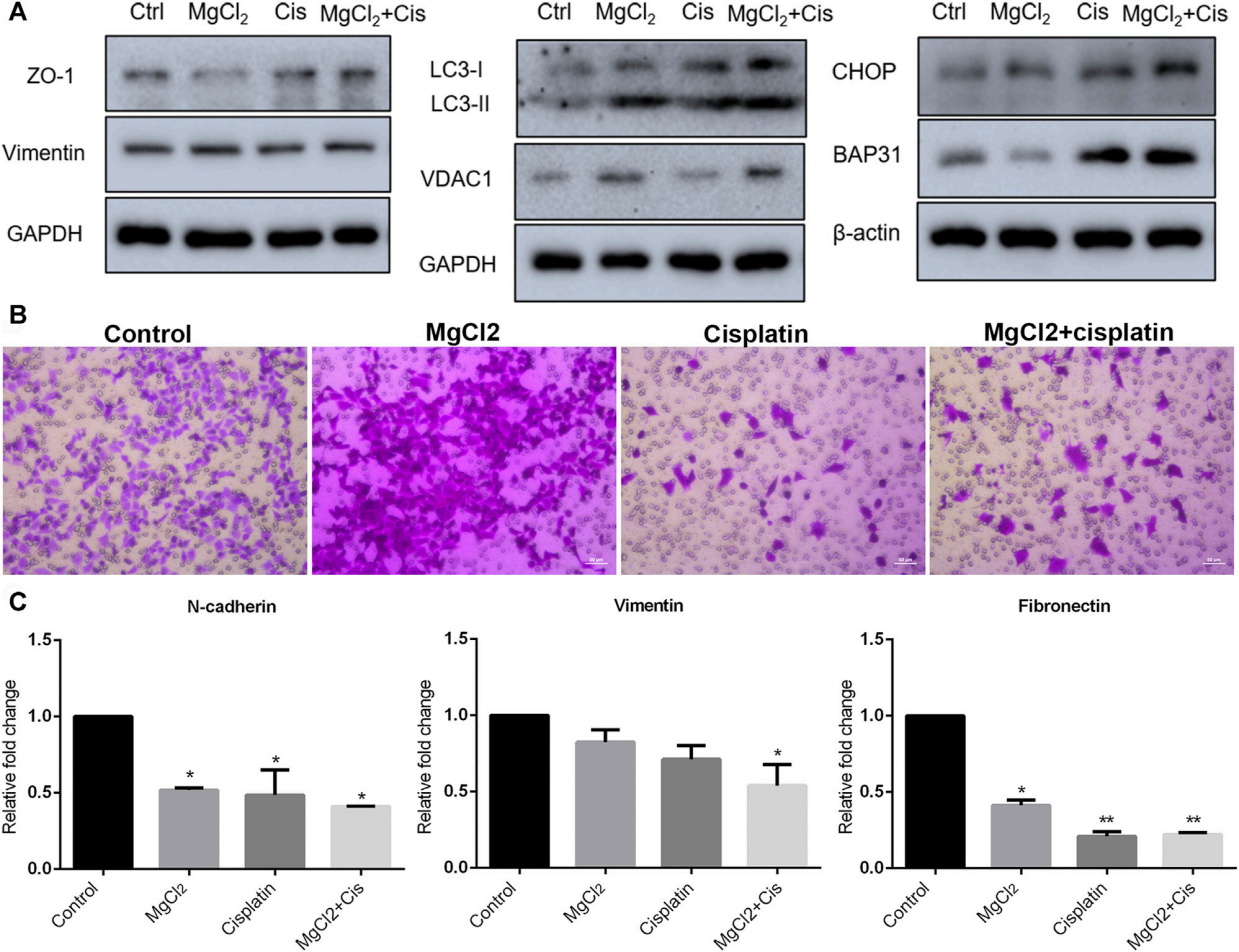
Migration of UC3 bladder cancer cells that received combinatorial treatment with MgCl_2_ and cisplatin (Cis). **(A)** Protein expression of migration-related genes as determined by western blot analysis. Densitometry of western blot was showed in Supplementary Figure S7A. **(B)** Cell migration was evaluated by the trans-well assay after cells treatment with MgCl_2_ and/or cisplatin for 24 h (also seen Supplementary Figure S7B). **(C)** mRNA expression of migration-related genes as examined by qPCR (mean ± SEM of duplicate experiments). *p < 0.05 vs. control and **p < 0.01 versus control.

Autophagy is a major cellular process of degradation that is critical for maintaining cellular homeostasis in response to various environmental stressors (Mathew et al., 2007; Lv et al., 2014). Autophagy has a dual role in cancer development in that it can either trigger cell death or promote tumor survival (Qu et al., 2003; Mathew et al., 2007). However, increasing evidence suggests that the modulation of autophagy could be a potential strategy for cancer therapy. Therefore, the induction of autophagy was evaluated in this study. The results of the western blotting indicated that the expression of LC3-II was up-regulated by drug treatment, especially in cells treated with the combination of MgCl_2_ and cisplatin (Figure 3A; Supplementary Figure S6). In addition, combinatorial treatment also enhanced VDAC1 expression. Endoplasmic reticulum (ER) stress, caused by the disturbance of ER homeostasis, is often closely linked to autophagy. The expression of CHOP and B cell receptor associated protein 31 (BAP31) was highest in cells that underwent combinatorial treatment with MgCl_2_ and cisplatin among the examined groups, indicating that ER stress was involved in the enhancement of the antitumor effect of cisplatin by magnesium. Generally, these results convey that the disturbance of cellular homeostasis in bladder cancer cells by cisplatin could be exacerbated through the induction of autophagy and ER stress by supplementing magnesium.

### 3.3 Migration of Bladder Cancer Cells is Suppressed by Combinatorial Treatment with MgCl_2_ and Cisplatin

Migration and metastasis, major characteristics of cancer, are shown to be closely associated with the establishment of drug resistance (van Staalduinen et al., 2018; Safa, 2020). To determine the role of magnesium in cisplatin-suppressed cancer cell migration, the transwell assay was performed. As shown in Figure 3B, there was nearly no difference in cells migration between the control group and the MgCl_2_-treated group. The relative mobility of cell treatment with MgCl_2_ was 0.9768 times compared to the control group. However, the number of migrated cells was greatly reduced in the cisplatin-treated group. Furthermore, the migration ability of cells that received combinatorial treatment with MgCl_2_ and cisplatin was also decreased. Compared to the control group, the relative mobility of cisplatin-treated group and MgCl_2_ combined with cisplatin treatment group was 0.040 and 0.069 times, respectively. The expression of N-cadherin, vimentin and fibronectin was examined by qPCR. The results showed that the expression of N-cadherin and fibronectin was downregulated in cells treated with MgCl_2_ alone, cisplatin alone, or the combination thereof. The expression of vimentin at the mRNA level was only decreased in cells that received the combinatorial treatment (Figure 3C). However, there was no significant difference in the expression of vimentin at the protein level among the examined groups (Figure 3A). As revealed by western blotting, the expression of ZO-1 was reduced in cells treated with MgCl_2_ but increased in cells treated with cisplatin. Importantly, the expression of ZO-1 was upregulated in cells that received combinatorial treatment with MgCl_2_ and cisplatin (Figure 3A; Supplementary Figure S6), further confirming that magnesium did not alter the inhibitory effect of cisplatin on migration.

### 3.4 Magnesium Enforces the Downregulation of Wnt/β-Catenin Signaling in Cisplatin-Treated Bladder Cancer Cells

**FIGURE 4:**
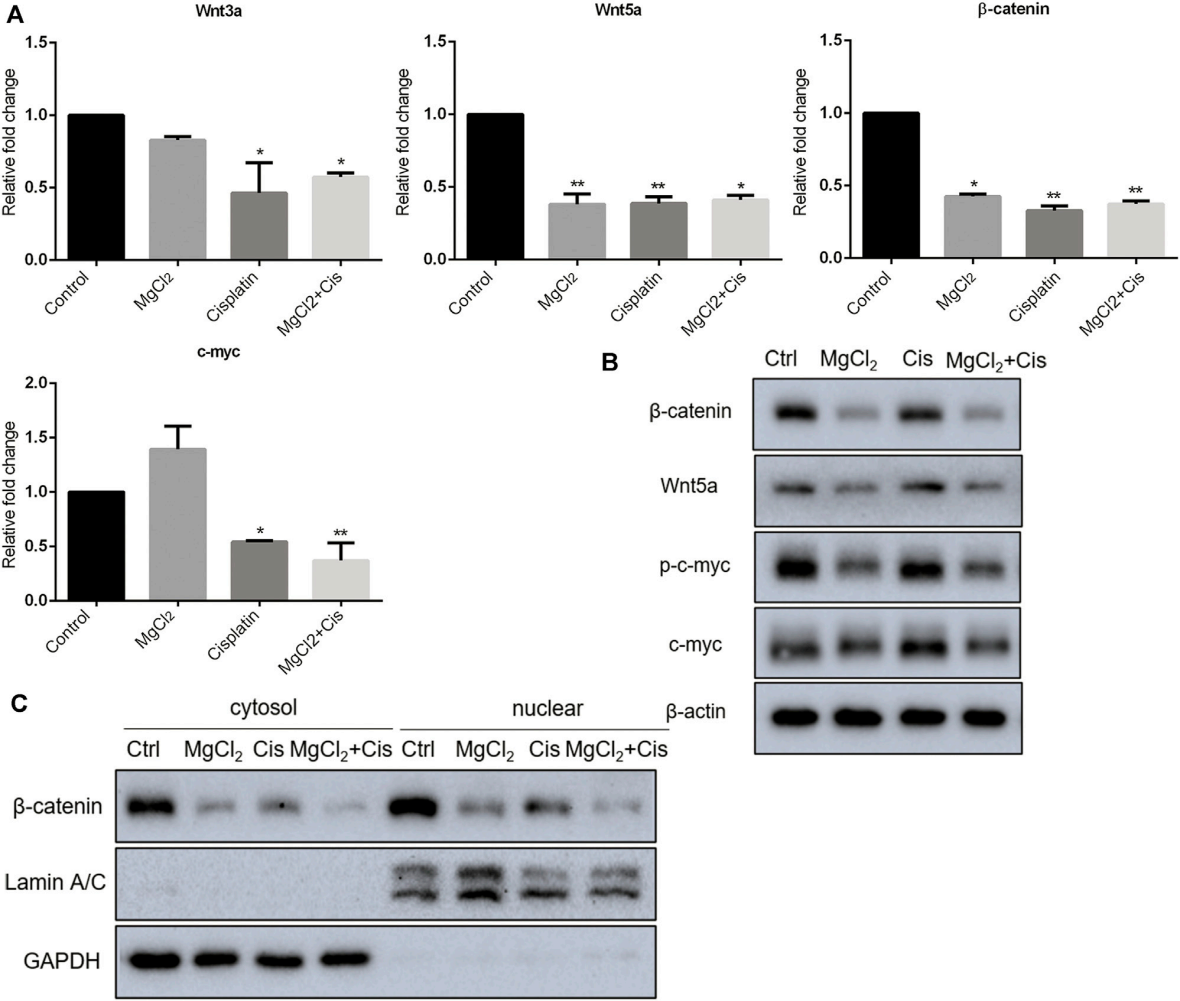
Expression of Wnt/β-catenin signaling components in UC3 bladder cancer cells received combinatorial treatment with MgCl_2_ and cisplatin (Cis). **(A)** Gene expression was determined by qPCR (mean ± SEM of duplicate experiments). *p < 0.05 vs. control and **p < 0.01 versus control. **(B)** Protein expression of Wnt/β-catenin signaling components and p-c-myc were assessed by western blot. **(C)** Protein expression of nuclear and cytosol β-catenin as evaluated by western blot. Densitometry of western blot was showed in Supplementary Figure S8.

**FIGURE 5:**
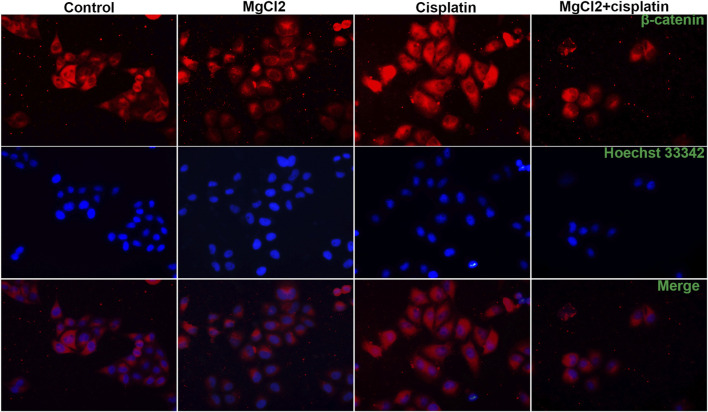
Distribution of β-catenin in UC3 bladder cancer cells that received combinatorial treatment with MgCl_2_ and cisplatin as determined by immunofluorescence. The immunofluorescence intensity was showed in Supplementary Figure S9.

Canonical Wnt/β-catenin signaling, a critical developmental signaling pathway, has attracted increased attention in the context of cancer chemotherapy (Zhang and Wang, 2020). In the present study, the results of the qPCR showed that the expression of Wnt3a and Wnt5a was decreased by combinatorial treatment with MgCl_2_ and cisplatin (Figure 4A). Western blot analysis confirmed that the expression of Wnt5a was downregulated by combinatorial treatment with MgCl_2_ and cisplatin (Figure 4B; Supplementary Figure S6). As for the core element of this pathway, the expression of β-catenin was decreased by combinatorial treatment with MgCl_2_ and cisplatin as revealed by both qPCR and western blotting. To further confirm the inactivation of Wnt/β-catenin signaling after MgCl_2_ and/or cisplatin treatment, nuclear protein was extracted and subjected to western blotting. There was no cyto-nuclear cross-contamination in nuclear protein. As shown in Figure 4C, the nuclear vs. cytosolic level of β-catenin was decreased in cells treated with MgCl_2_ and/or cisplatin, especially in cells that received MgCl_2_ treatment either alone or in combination with cisplatin. An immunofluorescence assay was also performed to further confirm the distribution of β-catenin. The results showed that MgCl_2_ treatment decreased the nuclear accumulation of β-catenin (Figure 5; Supplementary Figure S10). c-Myc is considered one of the target genes of the Wnt/β-catenin signaling pathway, and its expression was suppressed by MgCl_2_ treatment alone or in combination with cisplatin (Figures 4A,B). Furthermore, the phosphorylation of c-Myc was also decreased in cells received MgCl_2_ treatment or combinatorial treatment. These results indicate that magnesium could potentially inhibit Wnt/β-catenin signaling in cisplatin-treated cells (Figure 4B).

### 3.5 Activation of Wnt/β-Catenin Signaling Enhances the Inhibitory Effect of Magnesium on Bladder Cancer Cell Survival

**FIGURE 6:**
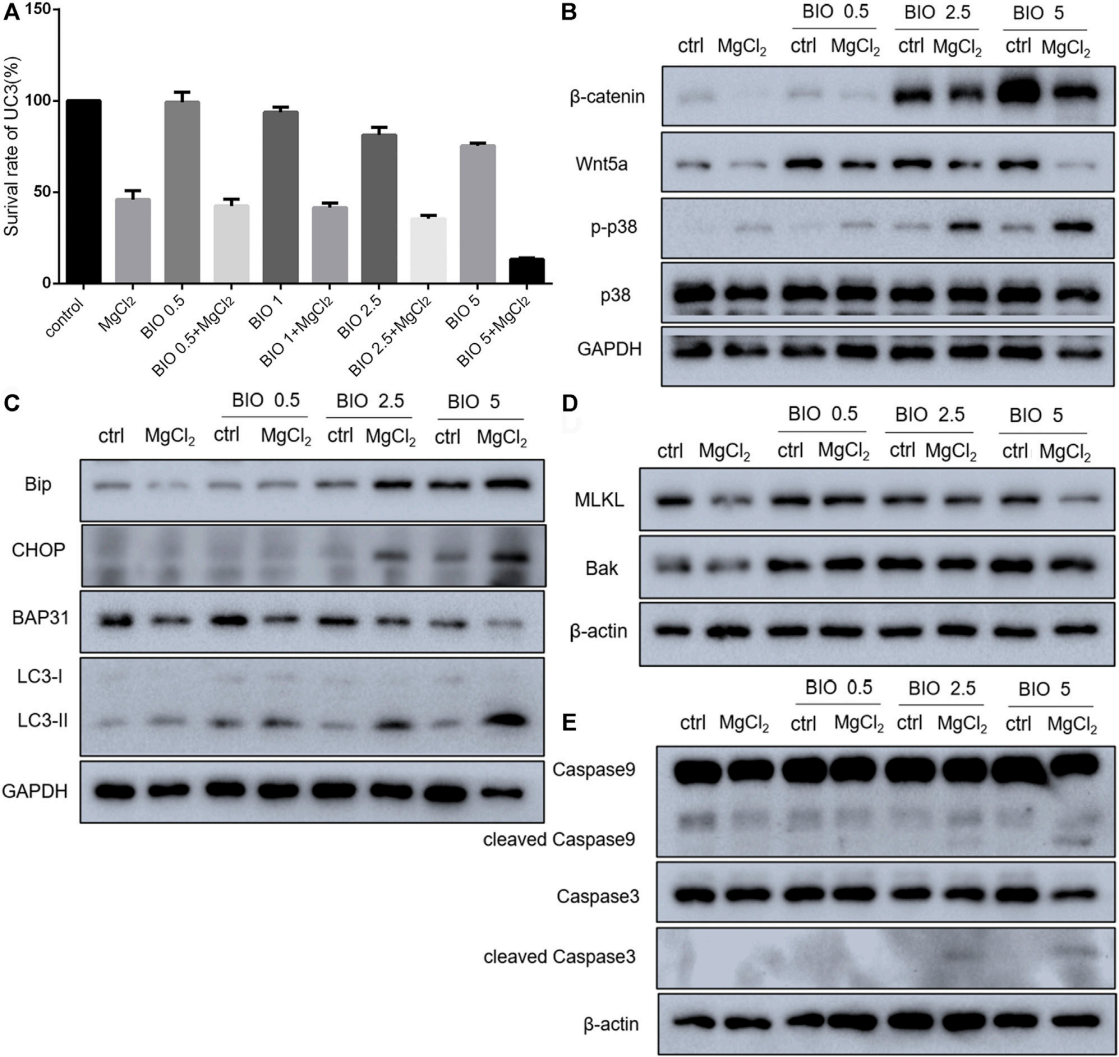
Effect of BIO treatment in MgCl_2_-treated UC3 bladder cancer cells. **(A)** The cell survival of UC3 bladder cancer cells treated with MgCl_2_ in combination with different concentrations of BIO was examined by the CCK-8 assay. **(B–E)** Protein expression of genes related to Wnt/β-catenin signaling components, the MAPK pathway, apoptosis, autophagy, and ER stress in MgCl_2_-and BIO-treated cells as determined by western blot analysis. Densitometry of western blot was showed in Supplementary Figures S11–S13.

**FIGURE 7:**
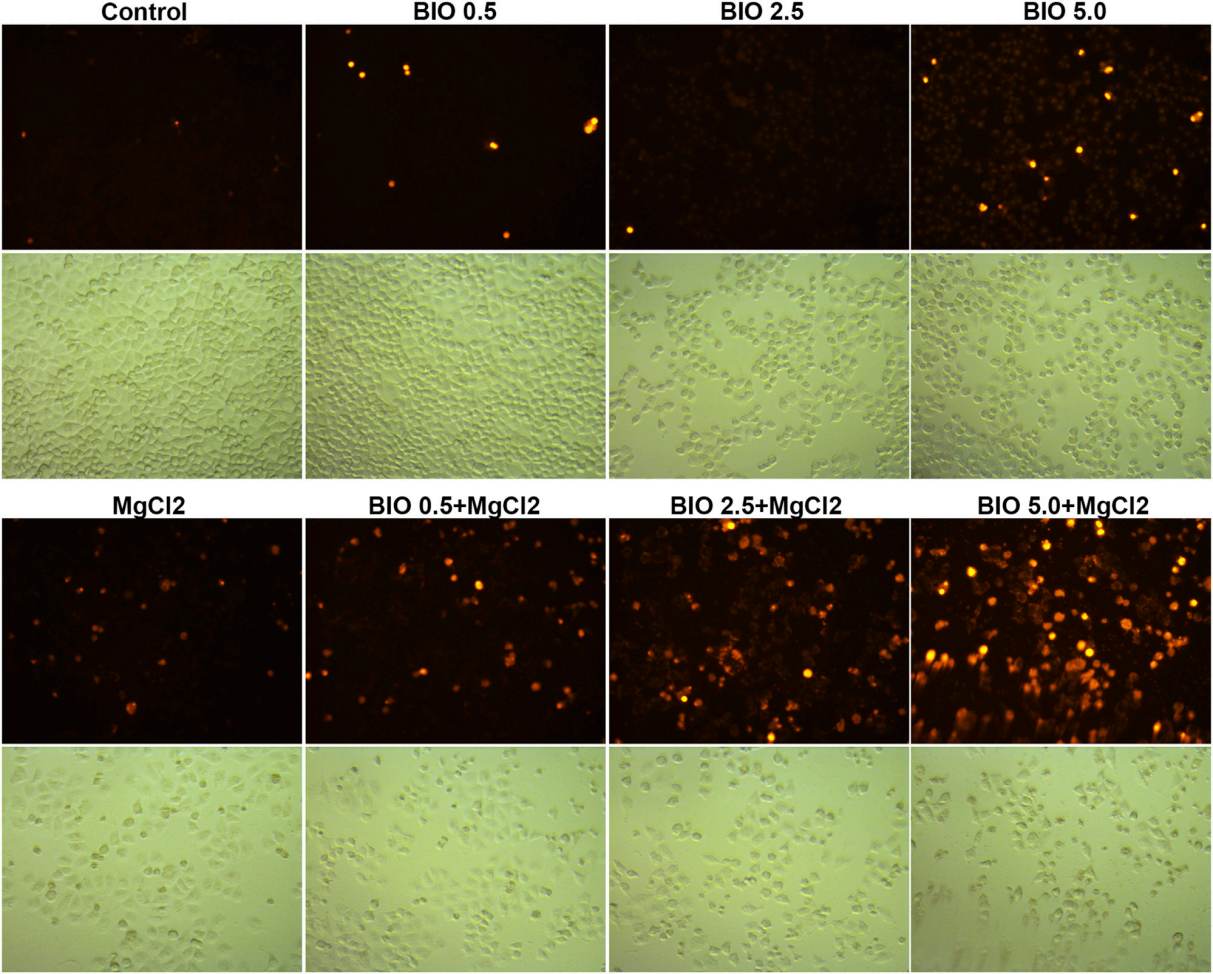
Determination of cell death in MgCl_2_-treated UC3 bladder cancer cells in combination with different concentrations of BIO treatment *via* PI staining. The immunofluorescence intensity of PI staining was showed in Supplementary Figure S14.

Considering that magnesium could inactivate Wnt/β-catenin signaling, as shown above, we decided to investigate the actual role of this signaling pathway in magnesium treatment. Therefore, the Wnt signaling pathway activator BIO was applied in the present study. The results of the CCK-8 assay showed that a concentration of 0.5 μM did not change the survival rate either in control cells or MgCl_2_-treated cells (Figure 6A). Meanwhile, western blot analysis indicated that the expression of β-catenin was only slightly enhanced by treatment with 0.5 μM BIO (Figure 6B). The survival rate of bladder cancer cells was decreased by BIO treatment at increased concentrations of 1.0, 2.5, and 5.0 μM (Figure 6A). In addition, 2.5 and 5.0 μM BIO induced the marked upregulation of β-catenin expression (Figure 6B). In both MgCl_2_-treated and untreated groups, the decreased survival rate was accompanied by increased β-catenin expression. However, the survival rate and β-catenin expression in MgCl_2_-treated cells were lower than those in MgCl_2_ untreated cells under the same concentration of BIO. Furthermore, the expression pattern of Wnt5a was similar to that of β-catenin in BIO treated cells (Figure 6B). Possibly, the magnesium-induced downregulation of Wnt/β-catenin signaling is in favor of cancer cell survival. To further confirm the role of Wnt/β-catenin signaling activation on cell survival, PI staining was performed. As showed in Figure 7 and Supplementary Figure S15, only a small number of cells suffered death after BIO treatment, and many more cells died when treated with MgCl_2_. As expected, BIO increased cell death at a dose-dependent manner in the MgCl_2_-treated cells.

Western blotting was performed to further investigate the molecular mechanism of magnesium treatment in the context of Wnt/β-catenin signaling activation. The expression of Bak was promoted by BIO treatment, and there was no significant difference between MgCl_2_-treated and untreated cells in terms of Bak expression (Figure 6D). The expression of cleaved caspase-3, was upregulated in cells received both MgCl_2_ and BIO treatment (2.5 and 5.0 μM, Figure 6E). Similarly, the expression of cleaved caspase-9 was also increased in the combinatorial treatment group with MgCl_2_ and BIO treatment (2.5 and 5.0 μM). These results indicate that magnesium-induced upregulation of apoptosis could be enhanced by the activation of Wnt/β-catenin signaling. The expression of MLKL in MgCl_2_-treated cells was lower than in control cells (Figure 6D). In MgCl_2_ untreated cells, BIO treatment (1.0, 2.5 and 5.0 μM) did not change the expression of MLKL. However, in MgCl_2_ -treated cells, the expression of MLKL was increased when cells were treated with 1.0 and 2.5 μM BIO. It seems that necroptosis was exacerbated in the condition where cells received combinatorial treatment with MgCl_2_ and low-concentration BIO. In MgCl_2_-untreated cells, there was no significant difference on the expression of LC3-II among the control, 1.0, 2.5 and 5.0 μM BIO groups (Figure 6C). However, the expression of LC3-II was enhanced by BIO treatment in a dose-dependent manner in MgCl_2_ -treated cells. This indicates that autophagy could be induced by combinatorial treatment with MgCl_2_ and BIO, rather than MgCl_2_ or BIO treatment alone. The expression of BAP31 was downregulated by MgCl_2_ treatment but unaltered by BIO treatment. The expression of Bip was elevated by 2.5 and 5.0 μM BIO and further promoted by MgCl_2_ treatment. The expression of CHOP was also enhanced by combinatorial treatment with MgCl_2_ and BIO (2.5 and 5.0 μM), suggesting that ER homeostasis in MgCl_2_-treated cells was further disturbed after Wnt/β-catenin signaling activation. The MAPK signaling pathway is closely associated with Wnt/β-catenin signaling. The results indicate that the phosphorylation of p38 MAPK was enhanced by BIO treatment in a dose-dependent manner and could be further increased by MgCl_2_.

## 4 Discussion

Uncontrolled cell proliferation is the hallmark of cancer. It has been well studied that cisplatin reduces cell proliferation by suppressing oncogenic pathways in various types of cancer (Achkar et al., 2018). However, the onset of chemoresistance and adverse side effects, including reduced immunity towards infections, nephrotoxicity, and hearing loss, are considered the main problems related to its clinical usage (Achkar et al., 2018; Giacomini et al., 2020). Combination therapy of cisplatin with other drugs is emerging as a promising means to overcome drug resistance and attenuate cytotoxicity. The present study confirmed that cisplatin could inhibit cell proliferation in both UC3 and UC5 bladder cancer cells. In addition, both MgCl_2_ and MgSO_4_ was found to strengthen the inhibitory effect of cisplatin on the survival rate of bladder cancer cells (Figure 1A; Supplementary Figure S2), suggesting that magnesium could play an important role in cancer treatment.

Programmed cell death, including apoptosis and autophagy, is closely involved in oncogenesis and metastasis (Fulda, 2010). Therefore, deciphering the signaling pathways underlying programmed cell death could aid the development of novel targeted antitumor therapeutic strategies. In this study, the results of the annexin V-FITC and PI staining indicated that the proportion of apoptotic and necrotic cells in cisplatin-treated cells was increased by MgCl_2_ treatment (Figure 2A, Supplementary Figure S4A). In addition, the ratio of Bax/Bcl-2 was also the highest in cells that received combinatorial treatment with MgCl_2_ and cisplatin among the examined groups, further confirming the enhanced pro-apoptotic role of combinatorial treatment (Figure 2A). Bax is a pro-apoptotic protein and Bcl-2 is an anti-apoptotic protein. It has been recognized that high ratios of Bcl-2/Bax often lead to poor outcomes with decreased rates of complete remission and low overall survival in cancer patients (Del Poeta et al., 2008). Additionally, the modulation of Bcl-2 and Bax family proteins using compounds has broad implications in cancer therapy (Pogmore et al., 2021). In this respect, magnesium may be conducive to strengthening the clinical usage of cisplatin. Certainly, the different forms of programmed cell death would jointly determine the fate of cancer cells. Autophagy is an evolutionary physiological mechanism that maintains cellular homeostasis in cells and may mediate autophagic cell death during development and pathogenesis (Schwartz, 2021). Growing evidence suggests that crosstalk between apoptosis and autophagy acts as a pivotal factor in cell fate determination. The results of this study showed that combinatorial treatment with cisplatin and MgCl_2_ promoted LC3-II expression, suggesting that autophagy had been induced (Figure 3A, Supplementary Figure S6). However, it should be noted that autophagy has a dual role in malignant cells, serving as both a tumor suppressor and promoter in a context‐dependent manner (Patra et al., 2021; Luo et al., 2021). Therefore, attempts should be made to utilize the anti-cancer role of autophagy. It has been shown that modulating the onset of autophagy could promote the antitumor effect of drug treatment (Huang et al., 2021b). Compared with cells that received MgCl_2_ treatment only, the survival rate was decreased but LC3-II expression was increased in cells treated with a combination of MgCl_2_ and BIO (Figure 6C). This indicates that enhancing the onset of autophagy could lead to higher rates of cell death among cancer cells. Autophagy can be triggered by many stress factors, including nutrient deprivation, ER stress, and hypoxia (White, 2015). The expression of CHOP was upregulated by cisplatin and MgCl_2_ treatment alone or in combination, indicating that ER stress was potentially involved in these treatments (Figure 3A, Supplementary Figure S6). Future study should also be performed to clarify the prominent cell death signaling pathway under combinatorial treatment with MgCl_2_ and cisplatin.

Wnt/β-catenin signaling is suggested as one of the main driving factors of various types of cancer (Wen et al., 2020). The results of the present study revealed that the decreased nuclear β-catenin could be further downregulated by combinatorial treatment with cisplatin and MgCl_2_ (Figure 4C, Figure 5, Supplementary Figure S6, Supplementary Figure S10). In addition, the expression of Wnt5a and c-Myc phosphorylation were also decreased in the combinatorial treatment group, further confirming that the Wnt/β-catenin signaling pathway was largely inhibited (Figure 4B, Figure 6C). Given its role signaling pathway in the initiation and progression of cancer, the Wnt/β-catenin signaling pathway is still being explored as a target for cancer therapy. In recent decades, several inhibitors, agonists, and antagonists have been developed to target this signaling cascade. BIO, a potent inhibitor of GSK3β that activates the Wnt/β-catenin signaling pathway, is found to reduce cisplatin nephrotoxicity without compromising its anti-proliferation function (Sun et al., 2020). In this study, BIO treatment was showed to reduce the survival rate of UC3 cells and strengthen the inhibitory effect of MgCl_2_ on cell proliferation (Figure 6A). In addition, levels of the cleaved forms of caspase-3 and caspase-9 were increased in cells that received both MgCl_2_ and BIO treatment (Figure 6E). It is well known that the caspase family can be divided into three main groups of proteins: initiator caspases (caspase-2, -8, -9, and -10) that trigger the caspase cascade; executioner caspases (caspase-3, -6, and -7) that are activated by initiator caspases and cleave a wide spectrum of cellular proteins; and inflammatory caspases (caspase-1, -4, -5, and -11) that participate in the regulation of an inflammatory response (McIlwain et al., 2015; Van Opdenbosch and Lamkanfi, 2019). Thus, it seems that BIO treatment contributed to enhancing the onset of apoptosis in MgCl_2_-treated cells (Figure 7). Several studies have found that the Wnt/β-catenin signaling pathway could serve as a bridge between apoptosis and autophagy during drug treatment (Reddy et al., 2020; Wu et al., 2020). In this study, activation of Wnt/β-catenin signaling by BIO could also promote the induction of autophagy (Figure 6C). However, it should be noted that the expression of β-catenin was lower in cells that underwent combinatorial treatment with BIO and MgCl_2_ than in cells treated with BIO alone (Figure 6B). Combined with the results showing that the expression of β-catenin was lower in cells treated with cisplatin alone, MgCl_2_ alone, and the combination thereof than in the control group, this possibly indicates that Wnt/β-catenin signaling contributes maximally to cancer therapy only when moderately activated. Therefore, the precise regulation of the Wnt/β-catenin signaling pathway to enhance magnesium based-cancer therapy should be exploited in future research.

## Data Availability

The raw data supporting the conclusion of this article will be made available by the authors, without undue reservation.
