# Supplementary material for: Magnesium-Assisted Cisplatin Inhibits Bladder Cancer Cell Survival by Modulating Wnt/β-Catenin Signaling Pathway
Source: Front Pharmacol. 2022 Jan 27;12:804615. doi: 10.3389/fphar.2021.804615 (PMC8829071; doi:10.3389/fphar.2021.804615)
Supplement: Supplementary file 1 [file Table1.PDF]

Supplementary Table S1. Primer sequences for qRT-PCR

Genes	Forward	Reverse
HRK	CCTACTGGCCTTGGCTGTG	TACAAGTTCCGCCTGCCG
TNFRSF10A	ACACCCAGCAAAGTGTGG	CCGACGACGACAAACTTG
TNFRSF10B	GACTATAGCACTCACTGGAATGACC	GTCATCGAAGCACTGTCTCAGAG
N-cadherin	GACGGTTCGCCATCCAGAC	TCGATTGGTTTGACCACGG
Vimentin	GACAATGCGTCTCTGGCACGTCTT	TCCTCCGCCTCCTGCAGGTTCTT
Fibronectin	TGACCTTTTCTGGCTCGTCT	GTTCAGCACAAAGGGCTCTC
Wnt3a	TGCATAGGCTCCTTCCTGTGG	TGGCTGGTGGGCTGAATTTC
Wnt5a	GAGTGCTCGCATCCTCAT	GCATGTCTTCAGGCTACA
β-catenin	GATTTGATGGAGTTGGACATGG	TGTTCTTGAGTGAAGGACTGAG
c-Myc	TCAGAGGTGCCACGTCTCC	TCTTGGCAGCAGGATAGTCCTT
GAPDH	GACAGTCAGCCGCATCTTCT	TTAAAAGCAGCCCTGGTGAC
