# Supplementary material for: Magnesium-Assisted Cisplatin Inhibits Bladder Cancer Cell Survival by Modulating Wnt/β-Catenin Signaling Pathway
Source: Front Pharmacol. 2022 Jan 27;12:804615. doi: 10.3389/fphar.2021.804615 (PMC8829071; doi:10.3389/fphar.2021.804615)

Magnesium-assisted cisplatin inhibits bladder cancer cell survival by modulating Wnt/β-catenin signaling pathway

Running Title: Magnesium in cisplatin treatment

Tianye Li [1], Zihan Tang [1], Chunting Li[1], Xiaoya Liu [1], Linglin Cheng[1], Zhijing Yang [2], Xiaojin Zhu [1], Weiwei Liu [2, 3], Yongye Huang [1, *]

[1] College of Life and Health Sciences, Northeastern University, Shenyang, 110169, China;

[2] Department of Oral and Maxillofacia Surgery, Hospital of Stomatology, Jilin University, Changchun, 130021, China;

[3] Jilin Provincial Key Laboratory of Tooth Development and Bone Remodeling, Changchun, 130021, China.

[*] To whom correspondence should be addressed: Tel: +86-24-83656116. Fax: +86-24-83656116. E-mail: huangyongye88@163.com (Yongye Huang).

Figure S1. Combinatorial treatment with $MgCl_2$ and cisplatin (Cis) suppresses the survival of UC3 bladder cancer cells. (A) The survival of cells treated with cisplatin under different concentration and time duration as determined by CCK8 assay. (B) Quantification for the colony formation of UC3 cells treated with MgCl2 and/or cisplatin. (C) The immunofluorescence intensity of PI staining was calculated as total IOD/total cell area. (D) Statistical analysis on Figure 2A based on Annexin V-FITC/PI staining cytometry.

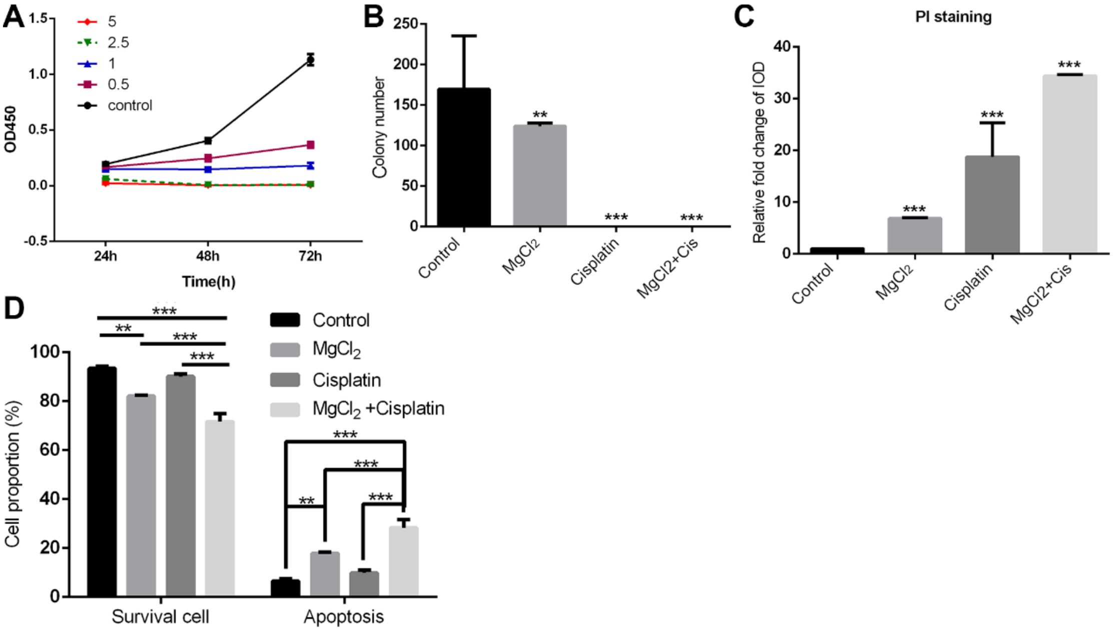

Figure S2. The survival of HEK293 and UC3 cells with different combinatorial treatment of cisplatin and magnesium for 24h as determined by CCK8 assay. 0.70 mM magnesium was recognized as 1×. *p<0.05 versus control, **p<0.01 versus control, and ***p<0.001 versus control.

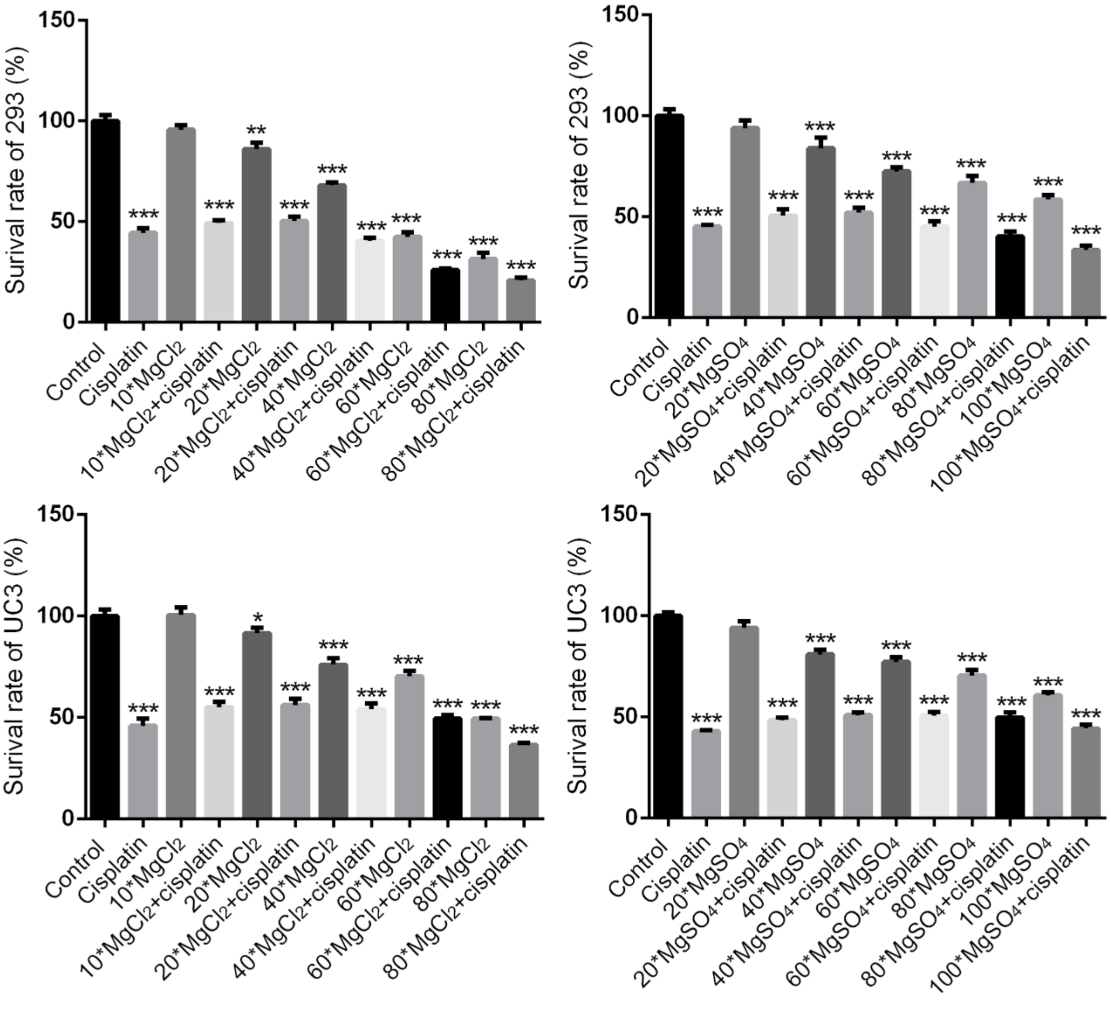

Figure S3. PI staining of UC5 cells treated with MgCl2 and/or cisplatin for 24 h.

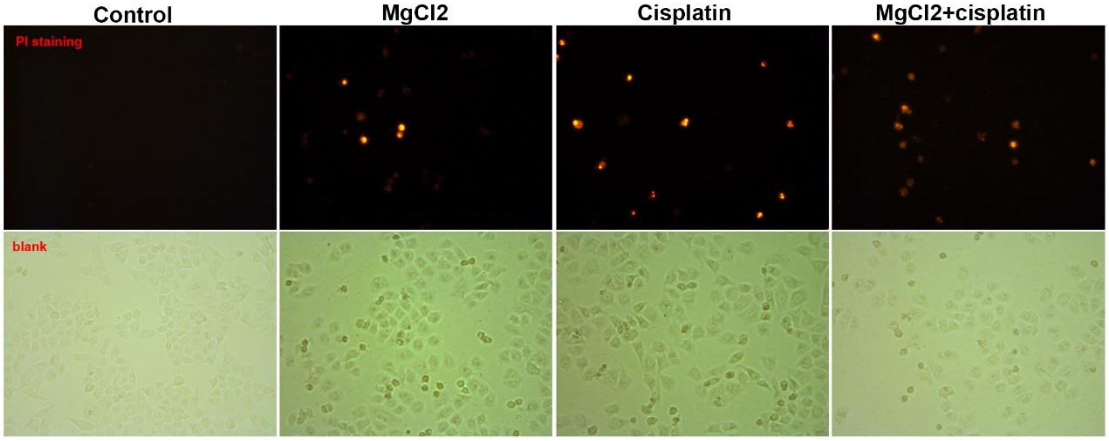

Figure S4. Combinatorial treatment with $MgCl_2$ and cisplatin (Cis) induces apoptosis in UC5 bladder cancer cells. (A) Apoptosis in bladder cancer cells treatment with $MgCl_2$ and/or cisplatin for 24 h was determined by annexin V-FITC/ PI staining using flow cytometry. (C) Expression of genes associated with apoptosis and cell cycle distribution as measured by western blot analysis.

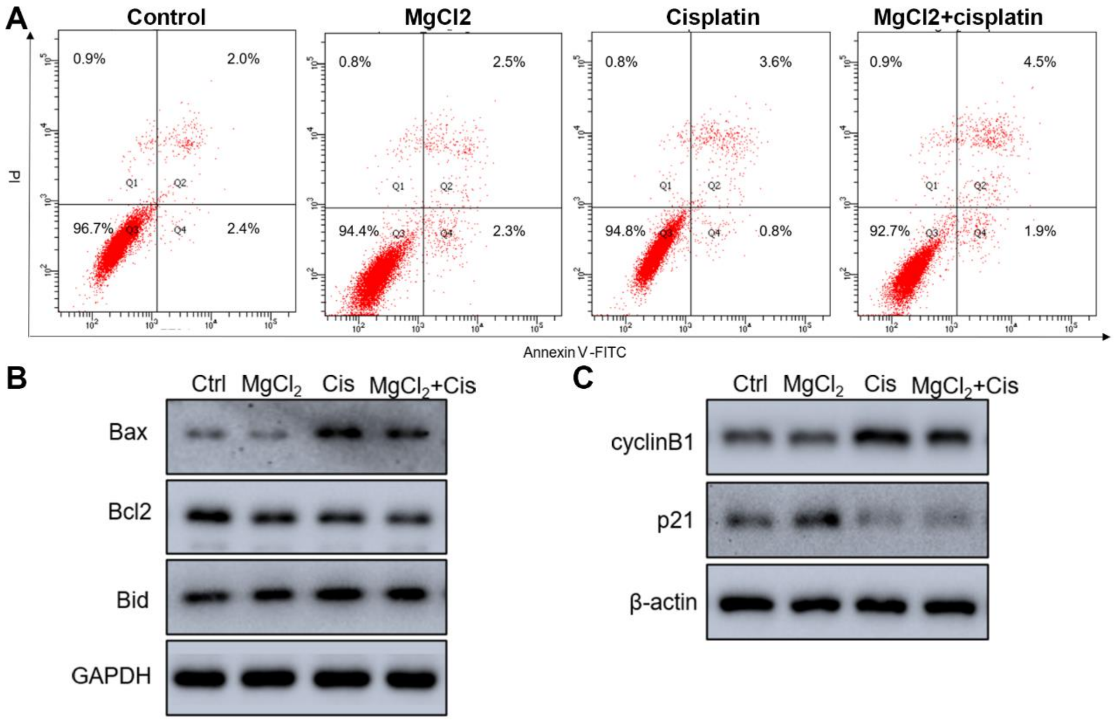

Figure S5. (A) Cell cycle distribution in bladder cancer cells was determined by PI staining using flow cytometry. (B-H) Densitometry of Figure 2C was calculated by comparing with internal protein.

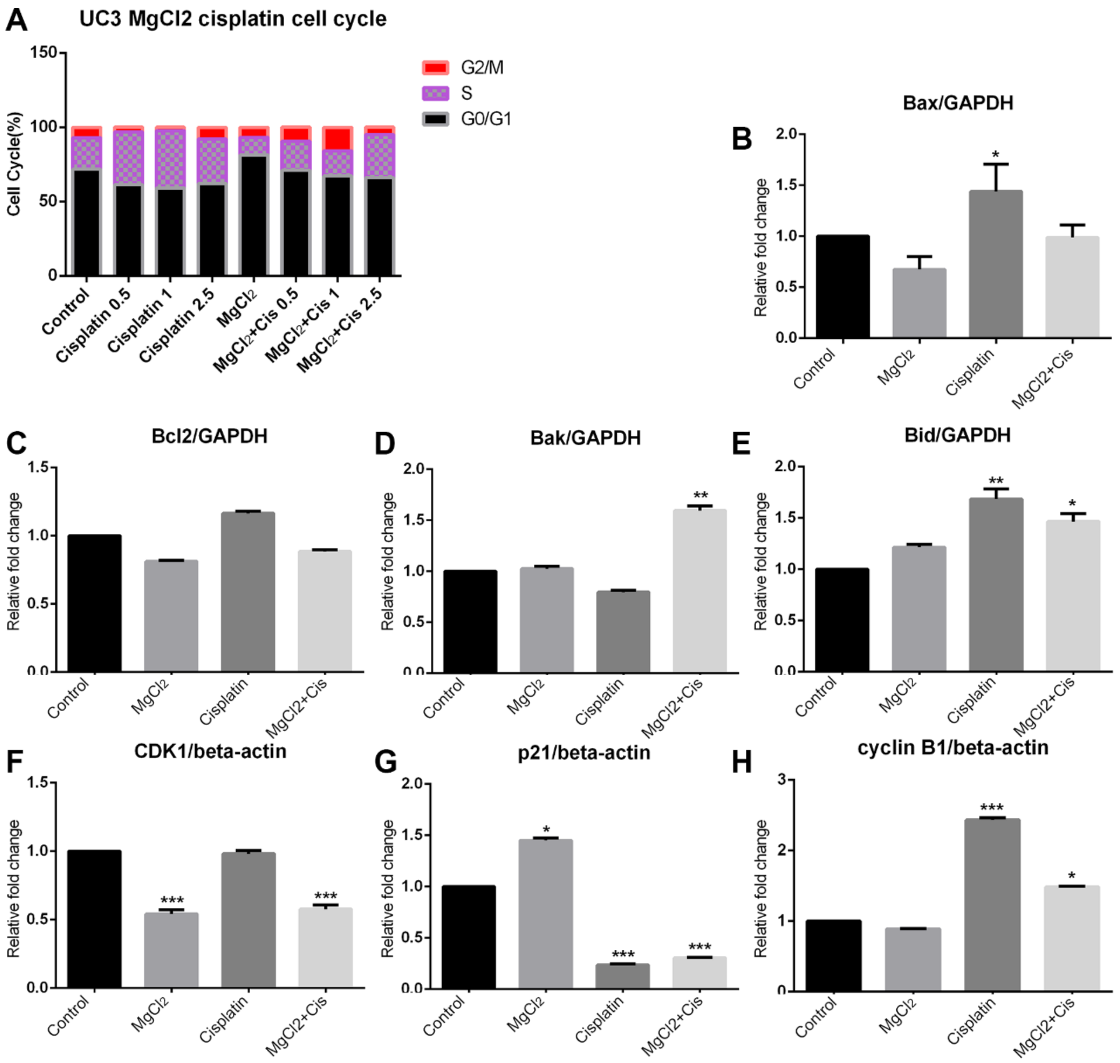

Figure S6. Protein expression in UC5 bladder cancer cells treated with MgCl2 and cisplatin (Cis) as determined by western blot analysis.

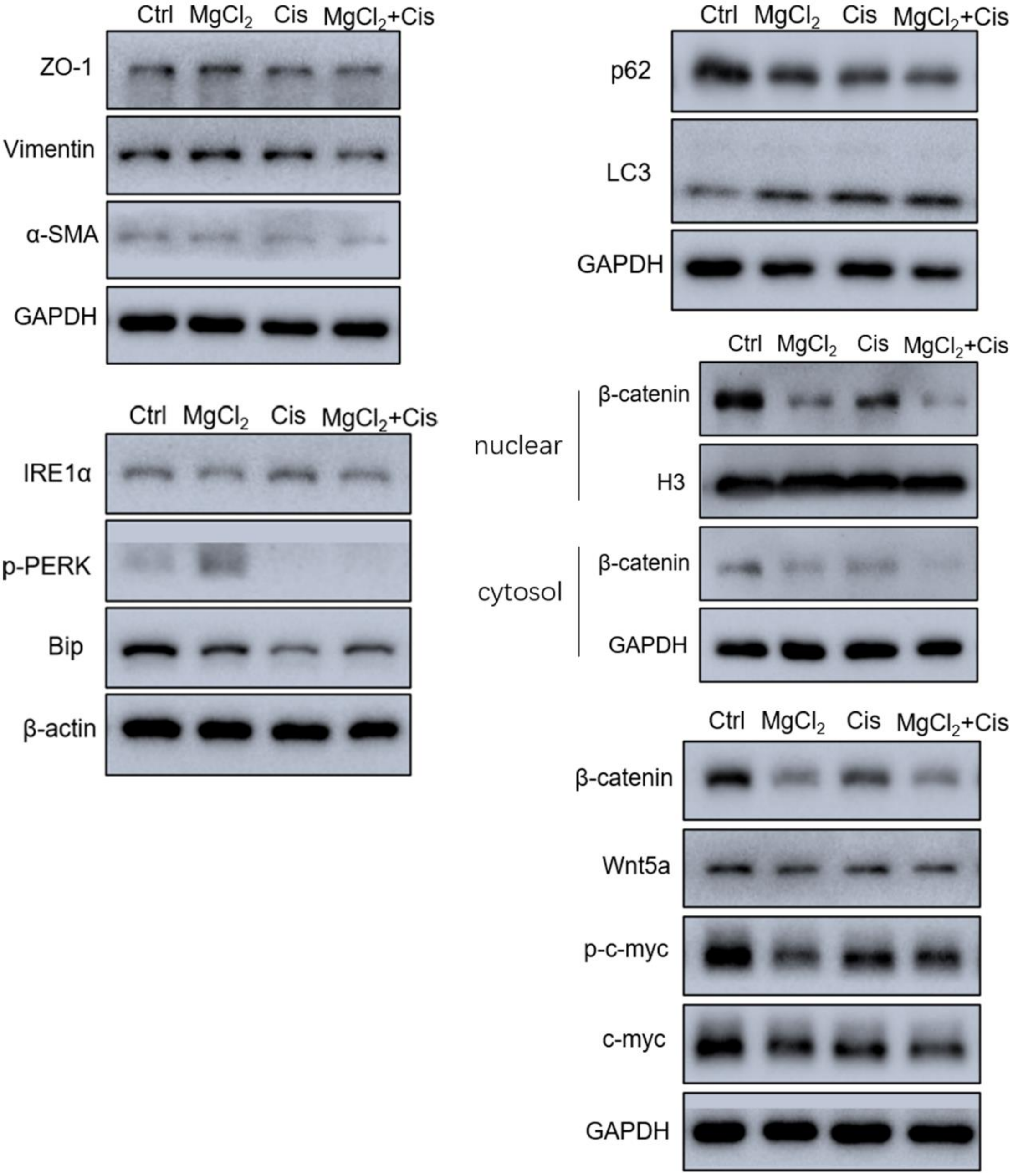

Figure S7. (A) Densitometry of Figure 3A was calculated by comparing with internal protein. (B) Statistical analysis on the migrated cell number based on transwell assay.

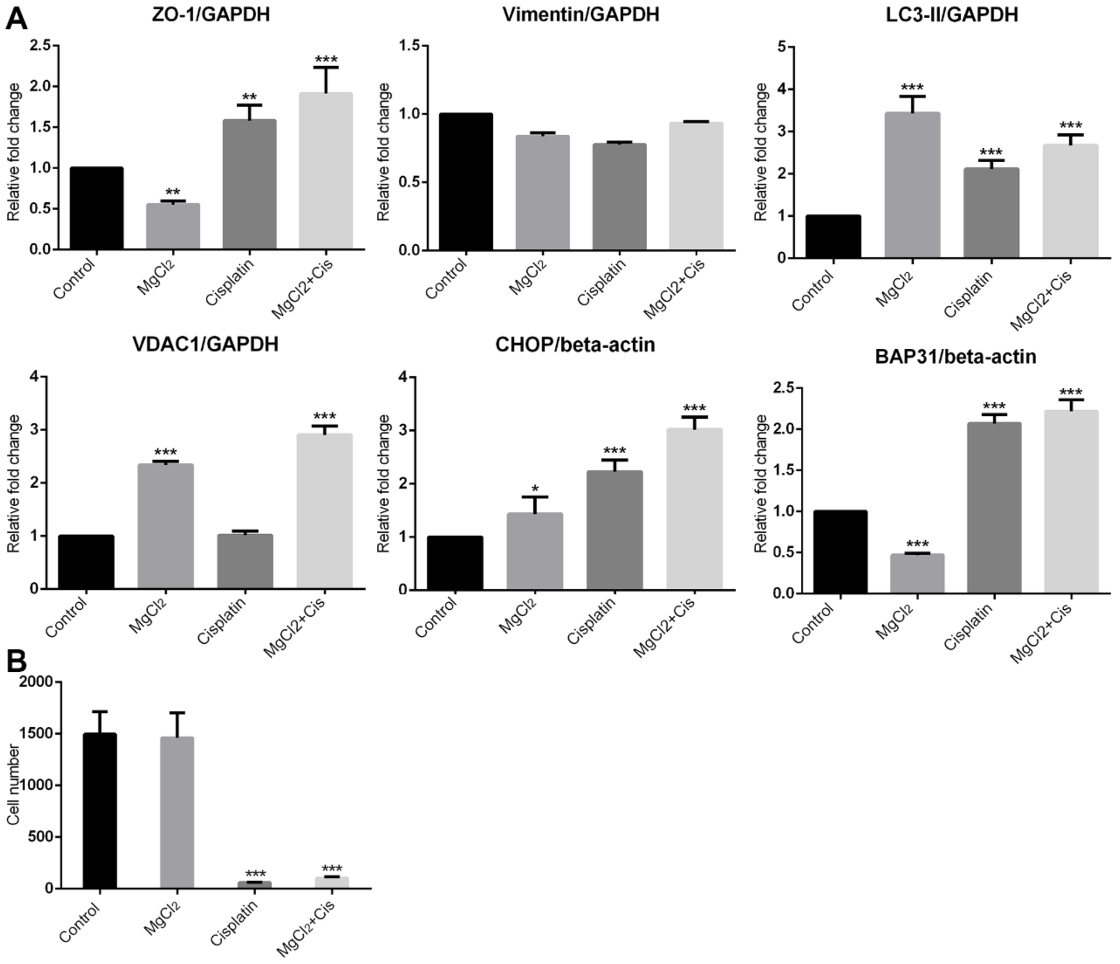

Figure S8. Densitometry in Western blot analysis was calculated by comparing with internal protein.

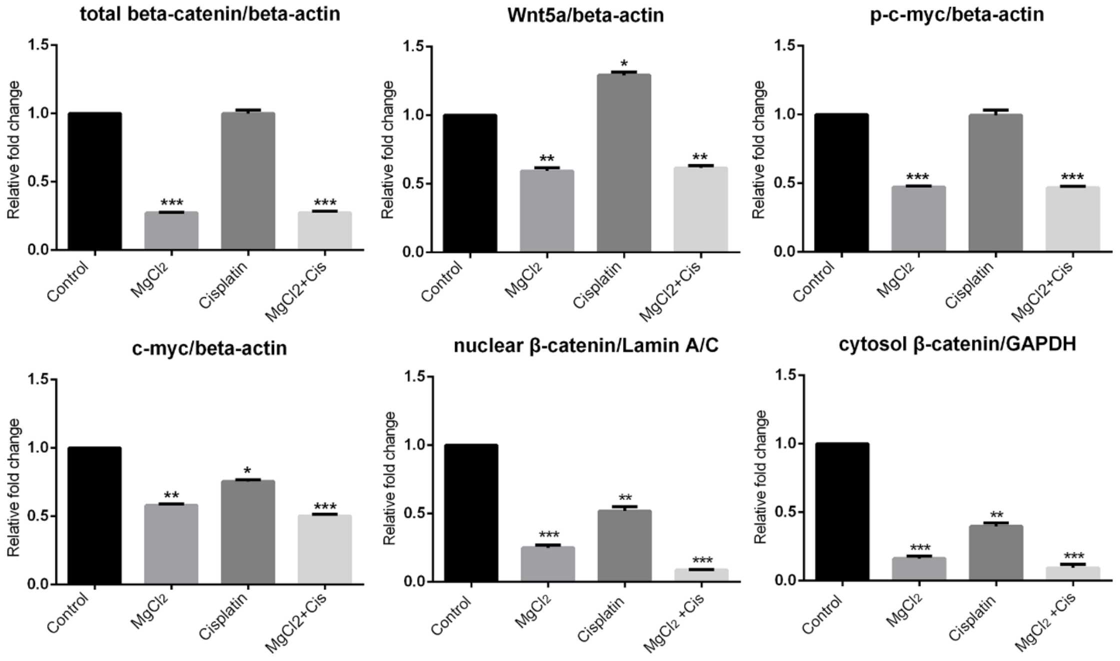

Figure S9. The immunofluorescence intensity was calculated as IOD/ area.

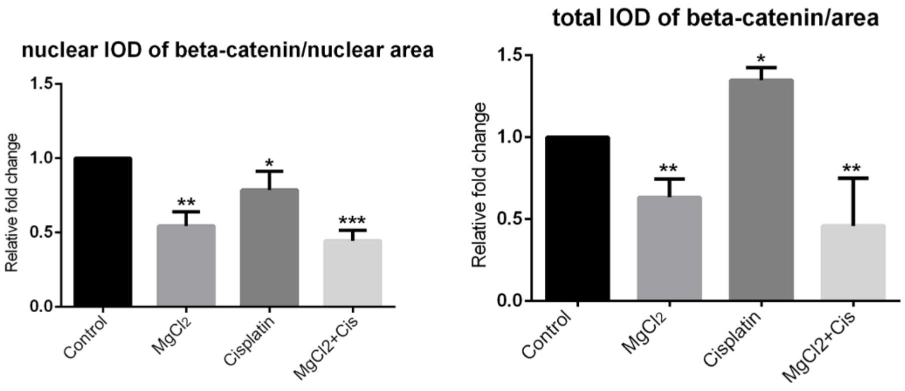

Figure S10. Distribution of β-catenin in UC5 bladder cancer cells that received combinatorial treatment with $MgCl_2$ and cisplatin as determined by immunofluorescence.

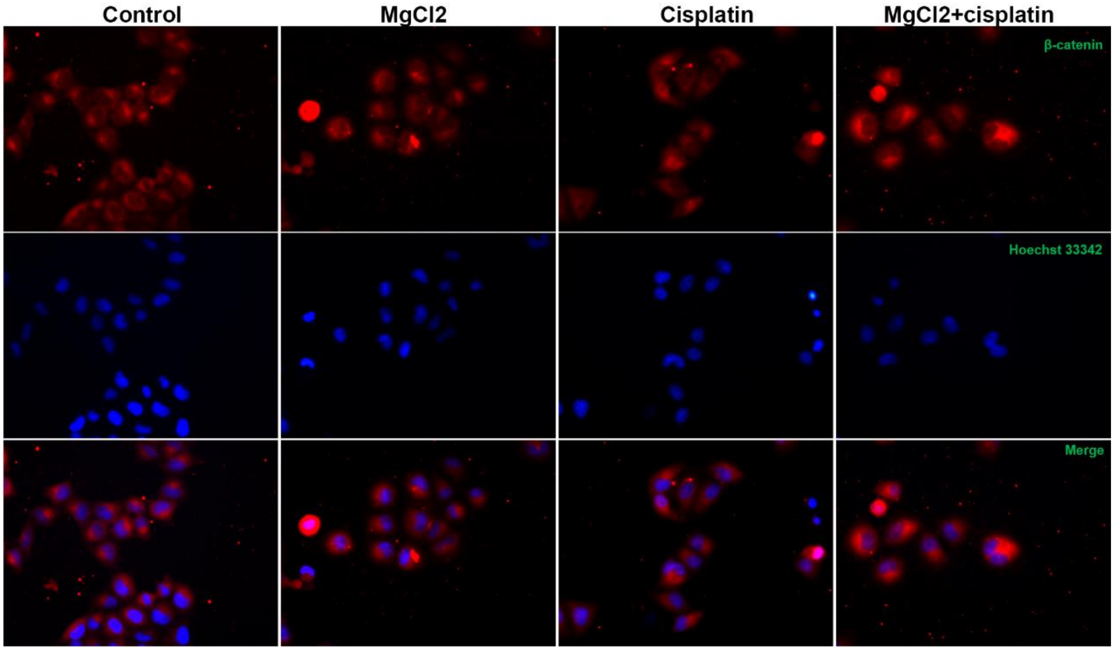

Figure S11. Densitometry of Figure 6B was calculated by comparing with internal protein.

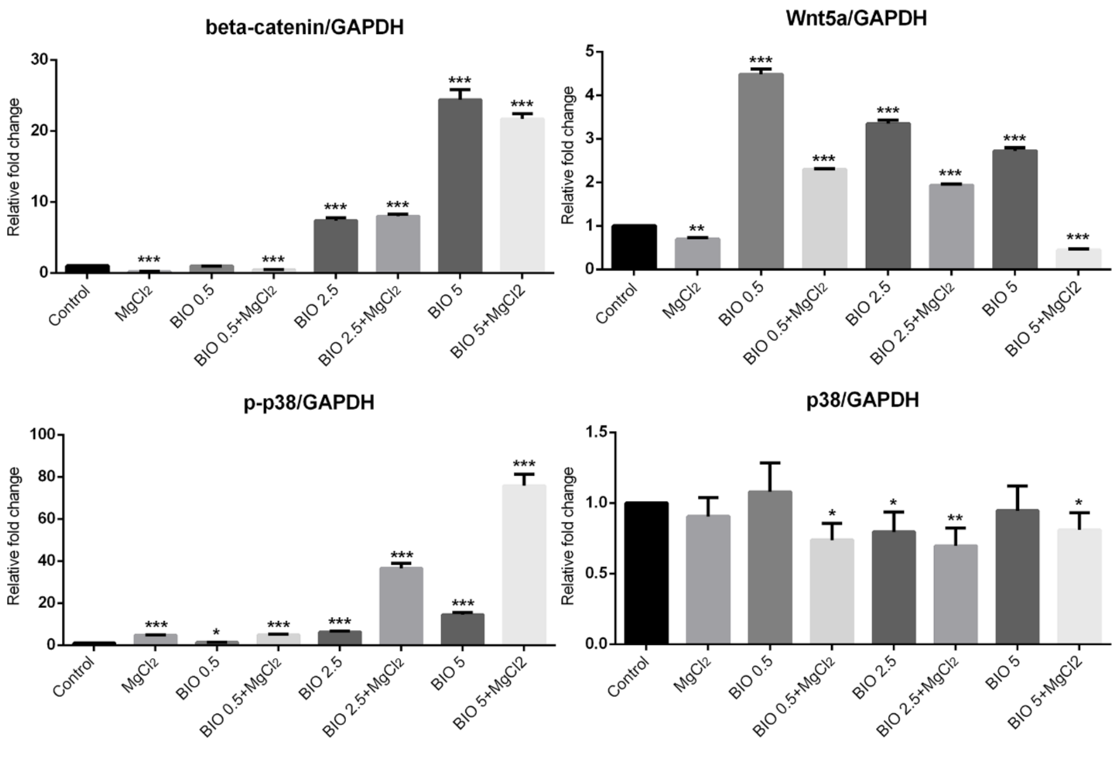

Figure S12. Densitometry of Figure 6C was calculated by comparing with internal protein.

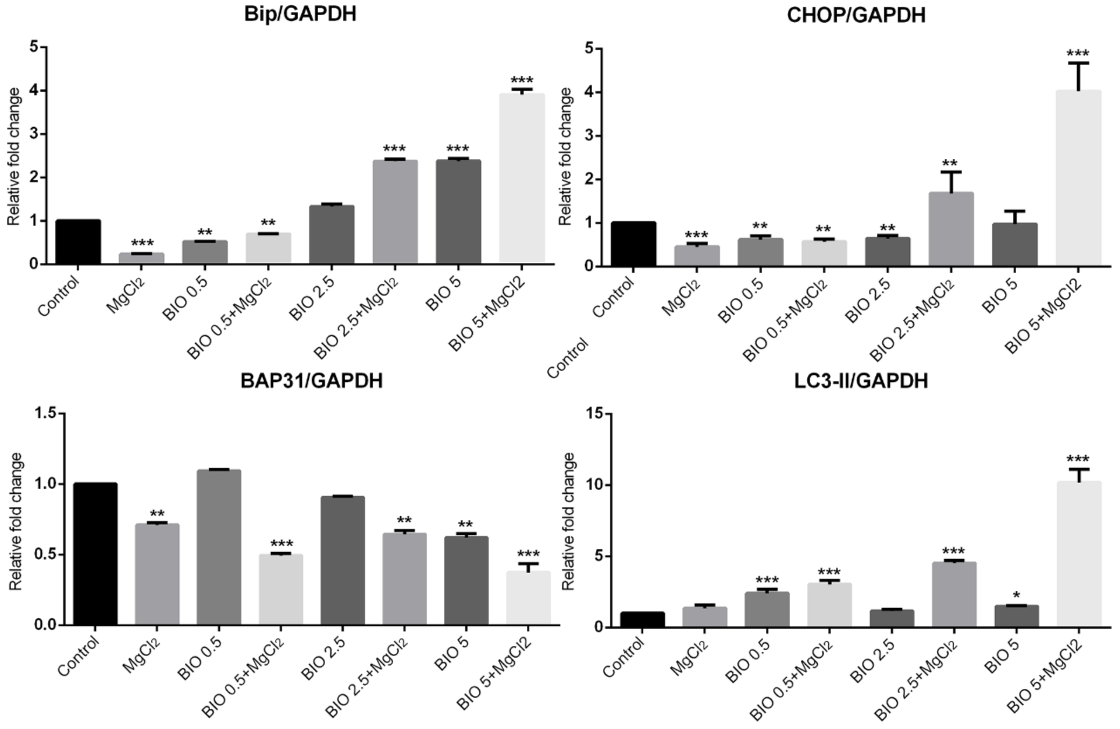

Figure S13. Densitometry of Figure 6D and 6E was calculated by comparing with internal protein.

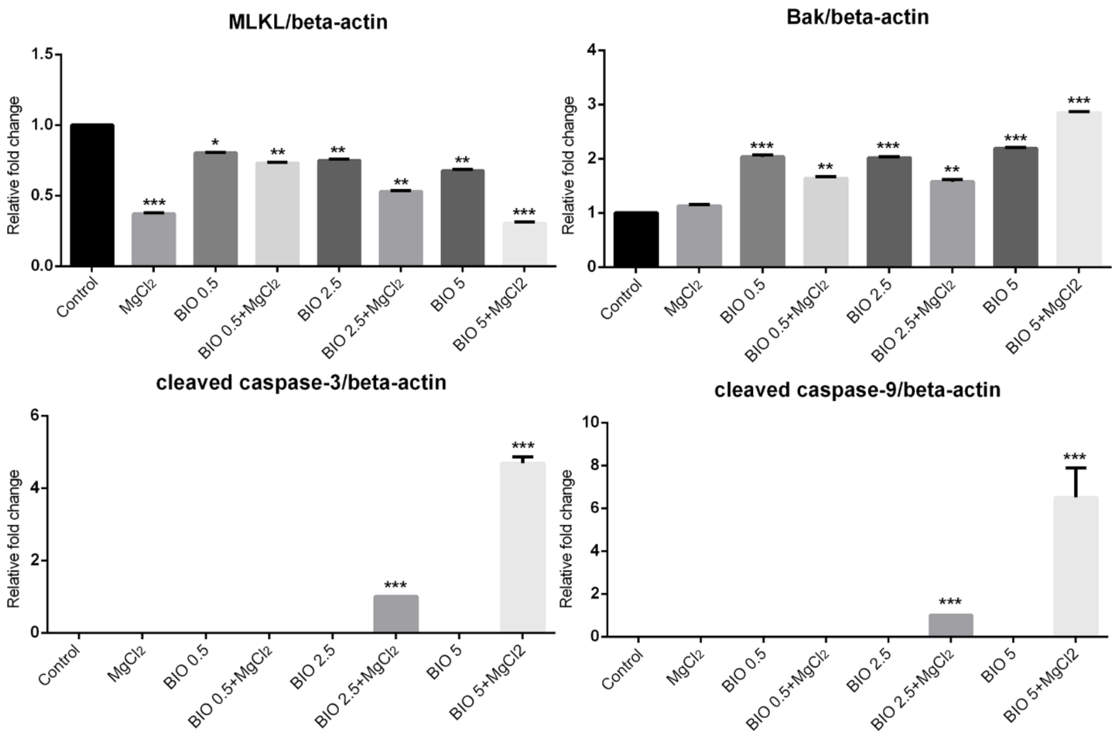

Figure S14. The immunofluorescence intensity of PI staining was calculated as total IOD/total cell area.

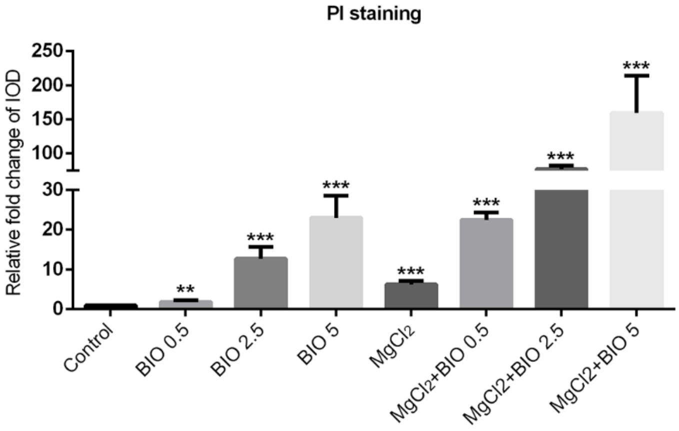

Figure S15. Determination of cell death in $MgCl_2$-treated UC5 bladder cancer cells in combination with different concentrations of BIO treatment via PI staining.

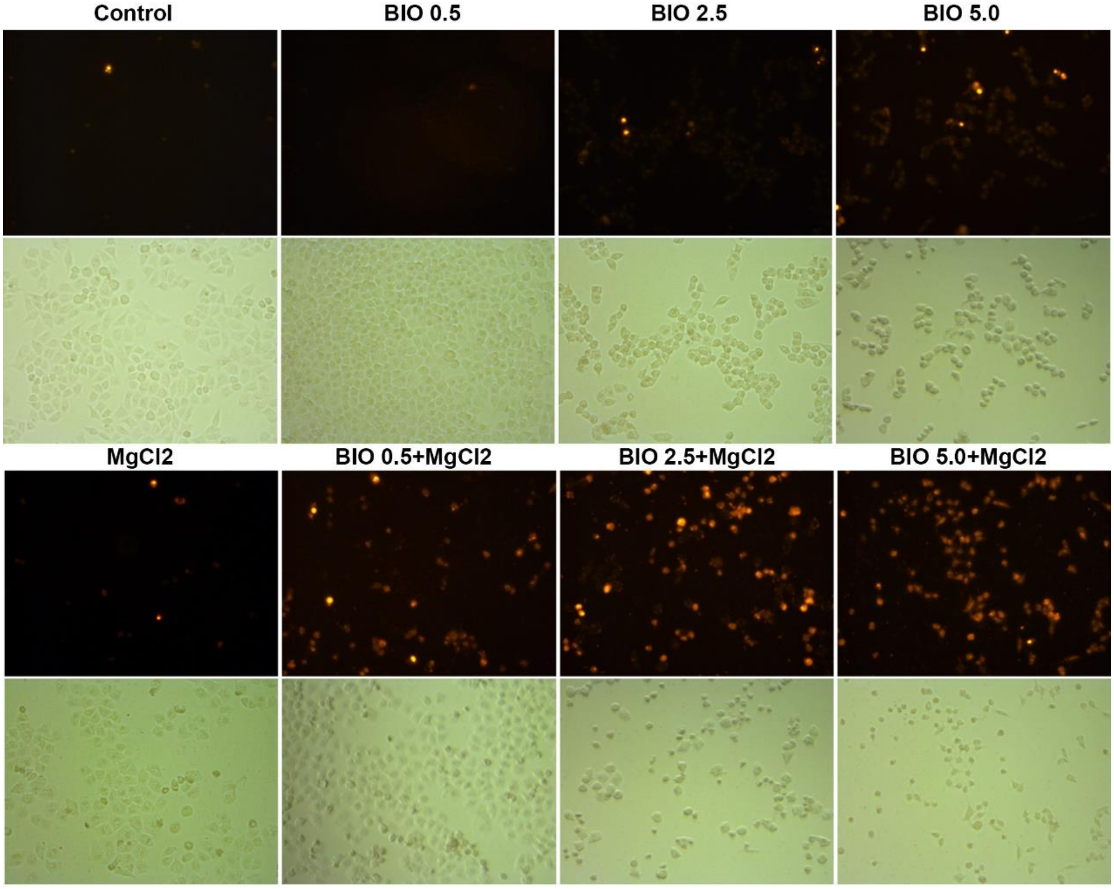

Supplement: Supplementary file 2 [file DataSheet1.pdf]
